# Risk Factors and Impact of Intra-Articular Scarring After Open Reduction and Internal Fixation in Mandibular Condylar Head Fractures—A Prospective Analysis

**DOI:** 10.3390/jcm14010266

**Published:** 2025-01-05

**Authors:** Clarissa Sophie Reichert, Simon Patrik Pienkohs, Linda Skroch, Axel Meisgeier, Andreas Neff

**Affiliations:** 1 Department of Oral and Maxillofacial Surgery, University of Marburg, Baldingerstrasse, D-35043 Marburg, Germany; 2 Clinic for Internal Medicine, Kreiskrankenhaus Bergstraße GmbH, Viernheimer Straße 2, D-64646 Heppenheim, Germany

**Keywords:** condylar head fracture, delayed ORIF, scarring, limitation of function, condylar resorption

## Abstract

**Background:** During the routine removal of osteosynthesis materials after surgical treatment (ORIF) of condylar head fractures (CHFs), as performed at our clinic, localised and sometimes pronounced intra-articular scarring were observed quite regularly. This prospective study therefore investigates the causes of intra-articular scarring and its impact on functionality after surgical treatment (ORIF) of condylar head fractures (CHFs). **Methods:** Moreover, 80/98 patients with 96/114 CHFs (ORIF between 2014 and 2024) were evaluated when performing hardware removal. Statistical analysis used logistic regression and sign tests. **Results:** Postoperative scarring was seen in 72/96 cases (75%), either localised (*n* = 54; 56%) or pronounced (*n* = 18; 19%). Scarring correlated with limitations of laterotrusion for pronounced scarring (*p* = 0.016; OR = 6.806; 95% CI [1.422, 32.570]; large effect size) and with limitations of mediotrusion for localised scarring (*p* = 0.013; OR = 0.236; 95% CI [0.076, 0.734]; very small effect size). Factors favouring localised scarring were reduced ipsilateral dental support (*p* = 0.022; OR = 3.36; 95% CI [1.191, 9.459]; medium effect size) and major fragmentation (*p* = 0.029; OR = 3.182; 95% CI [1.123, 9.013]; medium effect size). However, there was no correlation between scarring and types (screws w/wo microplates) or number of osteosynthesis materials. Pronounced scarring showed a significantly higher risk for osseous degenerative complications (*p* = 0.041; OR = 4.171; CI [1.058, 16.452]; medium effect size). **Conclusions:** Intra-articular scarring after ORIF of CHFs poses a risk for functional limitations and osseous degenerative changes. Early adhesiolysis during the removal of hardware seems favourable for functional outcomes after CHFs.

## 1. Introduction

**Figure 1:**
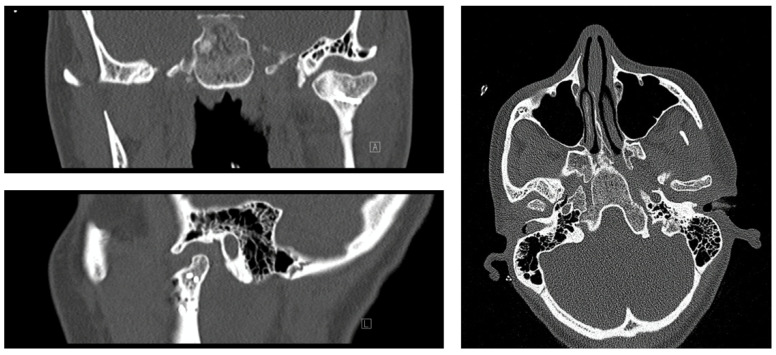
An 18-year old patient with vertical multilevel fracture of the condylar head and neck, 18 months p.o. (postoperative) with pronounced scarring of the inferior joint compartment and subsequent osteoarthrosis. Please note that some of the screws could not be removed due to osseointegration at the timepoint of the belated removal of the hardware.

Fractures of the condylar head (CHFs) are the second most common type of fracture of the mandibular articular process, accounting for 34% of all fractures [1]. Even though there is currently no uniform consensus on the treatment of such fractures [2,3], recent studies have shown that open reduction and internal fixation (ORIF) in CHFs reliably restores occlusion, and therefore reduces muscular problems and improves patient well-being due to better overall functional outcomes [2,4,5,6,7,8,9]. This is particularly true for bilateral fractures [10]. Nevertheless, the literature reports on possible postoperative mobility restrictions as a result of ORIF, which may arise from scar adhesions between the condylar surface and the disc, particularly in fracture types A and B, with type B fractures localised above or within the lateral capsular attachment zone, and type C below the lateral capsular attachment [11,12]. In the course of the routine removal of metallic implants (RMI) around 3–4 months after ORIF at the Department of Oral and Maxillofacial Surgery (OMFS), University Hospital Marburg, intracapsular scarring was repeatedly observed in patients with CHFs. Scarring was sometimes merely localised, sometimes also pronounced in extent. Localised scarring was found, especially in the temporomandibular joint’s (TMJ) dorsal recess or the lower joint compartment between the former fracture gap and the articular disc. Our working group reported in an earlier study about (minor) osseous resorption after ORIF during condylar remodelling and identified patient age and the degree of fragmentation of the CHF as factors favouring resorption [12]. Scarring was discussed as a possible factor affecting postoperative resorptive condylar processes [12] (see Figure 1). The fact that fibrous adhesions may contribute to functional impairments was already surmised in the literature [13,14]. Johner et al. investigated the direct influence of osseous resorptions on clinical function after removing the osteosynthesis material in this context; however, a direct correlation could not be demonstrated statistically by the working group [15].

Based on the results of previous studies by Johner et al. and Skroch et al., we hypothesised that intracapsular scarring has a major impact on bony resorption of the condylar head, as well as on postoperative function [12,15]. Accordingly, the aim of this study was to investigate the potential causative relationships of intracapsular scarring in the context of ORIF. Due to our intraoperative observations, we paid particular attention to the number and type of osteosynthesis materials used, as well as any protrusions and loosening of osteosynthesis material. We also hypothesised that there might be a correlation between fracture age and intra- and periarticular scarring, as relatively older condylar head fractures will incur a prolongation of the duration of surgery [16].

## 2. Materials and Methods

This study was approved by the Ethics Committee of the Department of Human Medicine of the Philipps University of Marburg, Baldingerstraße, 35043 Marburg, Germany (AZ: RS: 22/55, approved on 5 October 2022).

### 2.1. Study Sample

Inclusion criteria: Those eligible for this study were patients with displaced or dislocated mandibular condylar head fractures who underwent routine surgical treatment at the Department of Oral and Maxillofacial Surgery at UKGM GmbH, University Hospital Marburg between 06/2014 and 01/2024. The method of choice was open reduction and internal fixation (ORIF) involving at least two positional screws (SFPSO, MODUS CFS 1.8; Medartis, Basel, Switzerland), in some cases microplate osteosynthesis was applied for additional stabilisation, and the use of a retroauricular approach. Routinely, in all patients, the same surgeon performed a second procedure to remove the osteosynthesis material and, if necessary, for scar removal in the joint. Part of the sample (*n* = 41/96) was already included in a precursor study by Skroch et al. on factors favouring resorption during condylar remodelling after ORIF [12]. The working group led by Skroch et al. also developed the three-dimensional measurement method, which was also used in the present study to measure postoperative osseous volumetric changes in the condylar head [12].

Exclusion criteria: Patients with CHFs treated with surgical approaches other than the retroauricular approach were excluded from the study. Patients were also excluded if no second procedure was performed to remove the osteosynthesis material and therefore no intracapsular scarring was recorded.

### 2.2. Classification of Mandibular Condylar Head Fractures (CHFs)

The CHFs were classified according to the AO classificiation as non-fragmented, minor fragmentation, and major fragmentation, respectively, and, in addition to the AO classification, subclassified into fracture types A (type m of the AO classification), B, and C (type p of the AO classification) with type B fractures localised above or within the lateral capsular attachment, and type C below the lateral capsular attachment [11,12]. Multilevel fractures in which the articular process was involved, in addition to fractures of the condylar head, were included. Patients with bilateral CHFs requiring surgical treatment on one side only were categorised as unilateral fractures (e.g., contralateral non-displaced fracture of the condyle). Similarly, the condylar head fractures were categorised as unilateral for the purposes of this study if the anatomy was restored by ORIF in the presence of a contralateral articular process fracture (condylar base and/or neck fracture) or if there was no indication for surgical treatment (non-displaced fractures).

**Figure 2:**
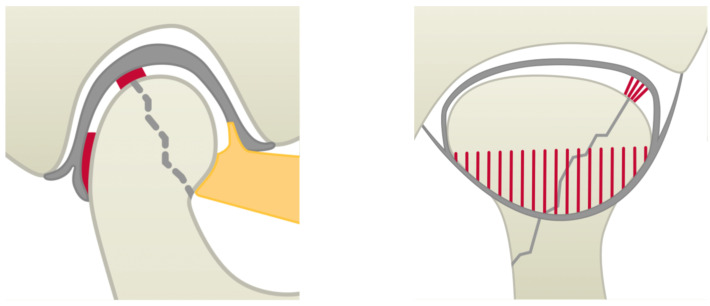
The lateral (**left**) and dorsal (**right**) view of the TMJ for a schematic visualisation of localised intra-articular scarring (red), occurring either between the former fracture gap (grey zigzag) and the disco-ligamental structures (grey) and/ or parts of the dorsal recess of the lower joint compartment; the lateral pterygoid muscle is shown as an orange area.

**Figure 3:**
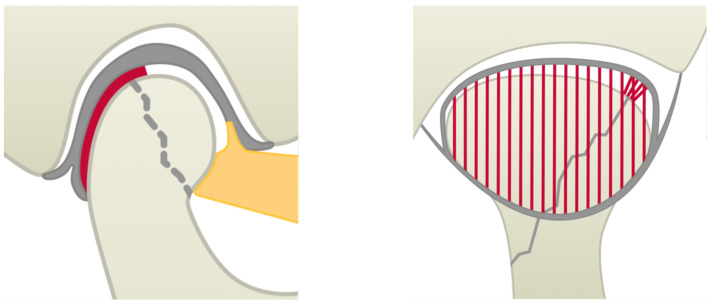
The lateral (**left**) and dorsal (**right**) view of the TMJ for a schematic visualisation of pronounced intra-articular scarring (red), here extending over the former fracture gap (grey zigzag) and the condylar surface and/or obliterating the dorsal recess of the lower joint compartment; the lateral pterygoid muscle is shown as an orange area and the disco-ligamental structures are grey.

The outcome variable defined was intracapsular scarring (subdivided into either localised or pronounced) at the time of the second procedure involving the removal of the osteosynthesis material (T2). Localised intra-articular scarring was defined to include isolated scarring in the temporomandibular joint (usually between the former fracture gap and the disc or in the lateral pole area in type B fractures) and incomplete fusion of the dorsal recess (see Figure 2). Cicatricial blockages and complete fusion, both in the dorsal recess and in the condylar surface region of the inferior joint space, were considered as pronounced scarring (see Figure 3). The classification of the degree of intra-articular scarring at T2 was documented by senior author A.N., who was active in the treatment of CHFs for 30 years, by intraoperative visual and haptic assessment.

### 2.3. Clinical Variables

A possible impact of patient-specific factors (age, gender, concomitant fractures, dental supporting zones to secure the vertical dimension present on the fracture side), the time elapsed until surgical treatment (T1) in days (fracture age), and the period between surgery (T1) and removal of metal removal (T2) in months on scar formation in the temporomandibular joint were investigated. Further clinical parameters recorded and examined in this study, in order to shed light on their significance for intra-articular scarring, were the osteosynthesis treatment selected (number of positional screws and microplates, defect augmentation with ß-TCP collagen fleece (Cerasorb Foam, Curasan AG, Frankfurt am Main, Germany)), changes in the postoperative course up to the time of removal of the metallic implant (T2) including volumetric changes in the condylar head and documented screw protrusions and/or loosening of osteosynthesis material (positional screws or microplates during material removal), possible complications (occurrence of temporary facial nerve palsies or wound infections), and osseous changes (osteophytes and/or heterotopic ossifications). The clinical range of function (protrusion, laterotrusion, mediotrusion, maximum mouth opening (MMO)) was also examined immediately prior to T2 during the preoperative patient education, and subsequently, in general, endotracheal anaesthesia (GETA) at T2 under objective conditions, categorised according to both the Clinical Dysfunction Index according to Helkimo and according to the Research Diagnostic Criteria (RDC/ TMD) [17,18], respectively (each categorised into the following: no, mild, moderate, or severe functional limitation, respectively) and numerically (in mm).

Originally, these indices are used for the diagnostics of temporomandibular disorders (TMDs), with TMD being a multifactorial disease affecting muskulosceletal structures of the head, especially the temporomandibular joint (TMJ), often referred to as arthrogenic TMD, primarily comprising arthralgia, the most frequent types of dysfunctions of the disc and osteoarthritis/osteoarthrosis [19,20,21]. Helkimo’s Index and the research diagnostic factors were developed to standardise the assessment and interpretation of functional metrics and bio-psychosocial risk factors for TMD [22,23,24], being objective parameters for easier diagnostics of this complex disorder. Helkimo categorised the assessment of TMD in two indices, namely the Clinical Dysfunction Index (D_i_) and the Anamnestic Dysfunction Index (A_i_). D_i_ surveys painful and limited range of movement in the mandible, functional impairments affecting the TMJ, and discomfort or sensitivity upon palpation of the (TMJ) and the masticatory musculature [17]. The Research Diagnostic Criteria are subdivided in two axes with a focus on clinical diagnostics (Axis I) and psychosocial anamnesis (Axis II). Axis I contains muscle and joint disorders, trauma, degenerative joint diseases, and collagen vascular diseases [18]. The present study exclusively used D_i_ and Axis I of RDC/TMD due to the focus on functional impairment. Nevertheless, considering the complex diagnosis of TMD, A_i_ and Axis II are essential [17,18].

### 2.4. Imaging and Volume Measurements

In order to survey osseous volumetric changes in the condylar head between ORIF (T1) and the second surgery with removal of the osteosynthesis material (T2) on the basis of CBCT and/or CT data as part of the postoperative controls after RMI, an eligible sub-sample of *n* = 92 condylar head fractures was defined as four fractures of the condylar head werenot completely depicted on the CBCT or CT scan at T1 or T2. Of these, *n* = 41/92 was already included and published in Skroch et al.’s study, whose three-dimensional measurement method was similarly applied in our study [12]. After importing the CBCT or CT data in the Digital Imaging and Communications in Medicine (DICOM) format and by applying a rendering, 3D models of the articular processes were created by Slicer freeware (open-source software of the National Institute of Health, Bethesda, MA, USA, version 4.20210226). The models were transferred to Blender freeware (Blender Foundation, The Netherlands, version 3.0.0 for Windows) in the form of stereolithographic data (STL) and the three-dimensional condylar head volume was measured. Volumetric changes in the condylar head between ORIF and metal removal were determined in absolute (in cm^3^) and relative (in %) terms. To verify the methodology according to Skroch et al. in the present sample [12], linear regressions were calculated, with the individual patient as a cluster variable due to interdependencies in the data. Relative and absolute volume changes (in cm^3^) were defined as outcome variables, and a possible statistical correlation with age, gender, and complexity of fracture (major/minor fragmentation) was examined.

### 2.5. Statistical Methods

Logistic regression models were calculated with cluster-robust standard errors and the patient as the cluster variable to allow for data interdependencies. The interdependencies resulted from the fact that some patients undergoing bilateral surgery, and both fracture sides were included in the analysis. The correlations were described using descriptive statistics (cross-tabulations), where the regressions could not be meaningfully analysed due to the small number of cases in the table cells. Due to the small number of cases, the models were calculated exploratively as individual bivariate analyses, not as multiple models.

Initially, localised and pronounced scarring was defined as an outcome variable, and a statistical correlation was examined with patient-specific data (age, gender, concomitant fractures), dental support present on the fracture side, fracture age in days, months elapsed between osteosynthetic treatment and RMI, fracture complexity (major/minor fragmentation), fracture type according to Neff et al. [11], the selected osteosynthetic treatment (number of positional screws and microplate osteosynthesis), defect augmentation with β-TCP collagen fleece, volumetric changes in the condylar head and documented screw protrusions and/or loosening of osteosynthesis material in the postoperative course, possible complications, and osseous changes (osteophytes and/or heterotopic ossifications). Furthermore, the impact of localised and pronounced scarring on the clinical range of function (protrusion, laterotrusion, mediotrusion, MMO) was examined, with intra-articular scarring also defined as an outcome variable.

In addition, the influence of fracture age on osseous changes was analysed as an outcome variable using logistic regression.

Possible correlations between volumetric changes in the condyle (both relative in % and absolute in cm^3^) with patient age, degree of fragmentation, gender, and osseous changes were tested using linear regression and excluding internal dependencies in the data due to bilateral CHFs, with the patient as a cluster variable.

When comparing function after surgical treatment and after scar adhesiolysis during the second procedure for metal removal, respectively, sign tests and two-sided t-tests were calculated, disregarding interdependencies in the data, as in the presence of a bilateral fracture, the functional range on either side will be exactly diametrically reversed in the patient (e.g., laterotrusion on the left = mediotrusion on the right).

The results were displayed in the form of odds ratios (ORs) or linear regression coefficients (B) and 95% confidence intervals (CIs). All statistical analyses were performed using Microsoft Excel^®^ 2023 (Microsoft, Redmond, WA, USA) and Stata (version 16.1, StataCorp LLC, College Station, TX, USA). A *p*-value ≤ 0.05 was defined as statistically significant. Interval-scaled data are described as mean ± standard deviation (SD) or median and ranges, and categorial variables as absolute and relative frequencies.

While the *p*-value indicates whether an effect exists, it does not provide information about the magnitude or practical relevance of the effect [25]. Therefore, we additionally reported the effect size (ES) to clarify the clinical relevance of our findings. To interpret the effect size (ES), we followed the categorisation of odd’s ratio described by Zieliński and Gawda, where an OR ≥ 1.44 indicates a small effect, an OR ≥ 2.48 indicates a medium effect, and an OR ≥ 4.27 indicates a large effect [26]. When interpreting effect sizes, it should be taken into account that the bigger the sample, the more likely it is to find statistical significant but eventually unimportant effects [25]. It is also important to note that while small effect sizes suggest a weaker association, they do not imply irrelevance; such findings may still hold clinical importance and warrant further investigation in future research. Also, medium and large effect sizes highlight more substantial effects, enhancing the robustness of the results and potentially guiding clinical recommendations.

Interval-scaled data are described as mean ± standard deviation (SD) or median and ranges, and categorical variables as absolute and relative frequencies. The sample size was calculated using GPower (version 3.1.9.7) to ensure adequate statistical power for detecting significant effects. The calculations were conducted based on an alpha level (α) of 0.05, a statistical power (1 − β) of 0.8, and two-tailed tests [27]. An effect size of g = 0.25, classified as large according to Cohen, was assumed [28]. The analysis indicated that a minimum sample size of 96 condylar head fractures would be required to achieve sufficient power. Due to expected drop-outs, our original sample decreased from 114 CHFs to a final sample including 96 fractures. For specific analyses involving subsets of the data—such as sign tests—the effective sample size varied due to the exclusion of tied observations. This adjustment resulted in reduced sample sizes for these analyses (e.g., *n* = 37 for the comparison of preoperative and postoperative Helkimo index scores).

## 3. Results

Between 06/2014 and 01/2024, 114 patients with condylar head fractures underwent surgical treatment at the Department of Oral and Maxillofacial Surgery at UKGM GmbH, University Hospital Marburg. Of these, 34 patients had to be excluded from the study for the following reasons: complete 3D imaging was not available at the time of surgical treatment (T1) and/or at the time of RMI (T2) in 14 cases; surgical treatment was not possible with positional screws due to the complexity of comminution in two patients, who were treated with plate osteosynthesis instead; and a second procedure and metal removal was not performed in 18 patients.

Moreover, 80 patients with a total of 96 condylar head fractures were included in the sample, of whom 44 were females (55%) and 36 were males (45%). A total of 16 patients (20%) exhibited bilateral condylar head fractures (9 females (11.25%), 7 males (8.75%)). The age at the time of surgery ranged between 14 and 85 years (mean = 46.18; SD = 19.09; Med = 48.5) (see Table 1). The osteosynthesis material was removed after an average of 3.97 months (SD = 2.05; range 2–16 months; Med = 3).

There was no statistically significant correlation between localised or pronounced intra-articular scarring in the temporomandibular joint and age, gender, or concomitant fractures on the fracture side (see Table 2 and Table 3).

Loss of the posterior dental supporting zone on the fracture side showed a statistically significant positive correlation with partial intra-articular scarring (*p* = 0.022); the effect size was medium according to Zieliński and Gawda’s categorisation [26].

No statistically relevant effect on intra-articular scarring could be confirmed as well as the number and type of osteosynthesis materials used (positional screws with/without microplate osteosynthesis), screw protrusions, and loosening (see Table 2).

In contrast, the complexity of the fracture on intra-articular scarring was shown to have a statistically significant impact in the analysis at hand, in the form of a correlation between the presence of the fracture with major fragmentation (according to the AO classification of 2014) and localised intra-articular scarring (*p* = 0.029; OR = 3.182) with a medium effect size [12,26]. However, minor fragmentations did not significantly affect the formation of adhesions in the temporomandibular joint. A correlation between postoperative volumetric changes in the condyle, both relative and absolute, and localised scarring could also not be verified statistically (see Table 2).

**Figure 4:**
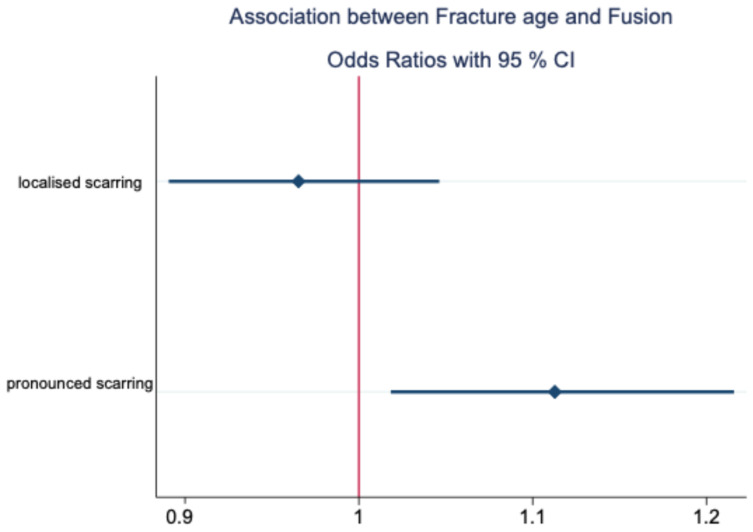
Association between fracture age and localised (i.e., partial)/pronounced (i.e., subtotal) fusion.

In fractures treated with delay, scarring was more pronounced (*p* = 0.018); however, the effect size describing this connection was small (see Table 3). The earlier the fracture was treated, the lower the risk of a higher degree of limitation and/or subtotal fusion in the dorsal recess or in the lower joint compartment, in the meaning of pronounced scarring (see Figure 4 and Table 3). In addition, there was a statistically significant correlation between postoperative osseous changes and pronounced scarring (*p* = 0.041), underlined by a medium effect size (see Table 3) [26].

In addition, an association was shown between fracture age and postoperative osseous changes with *p* = 0.002 (OR = 1.167; 95% CI = [1.059, 1.286]; very small effect size) [26]. The odds ratio of OR = 1.167 illustrated that the likelihood of heterotopic ossification or osteophyte formation present at the time of removal of the metallic implant increased by a factor of 1.167 with each day that elapsed from trauma until ORIF; however, the effect size was very small.

The data showed a correlation between function at the time prior to removing the metallic implant and intra-articular scarring. Patients with a higher degree of limitations and pronounced fusions in the lower joint space or dorsal recess were significantly more likely to exhibit a laterotrusion of ≤3 mm (*p* = 0.016; OR = 6.806; 95% CI = [1.422, 32.570]), substantiated by a large effect size, according to Zieliński and Gawda [26]. Localised scarring showed a significantly lower probability of limited mediotrusion ≤ 5 mm (*p* = 0.013; OR = 0.236; 95% CI [0.076, 0.734]) with a very small effect size [26]. When categorising the function according to Helkimo or according to RDC/TMD [17,18] correlations with intra-articular scarring could not be statistically proven (see Table 4).

**Figure 5:**
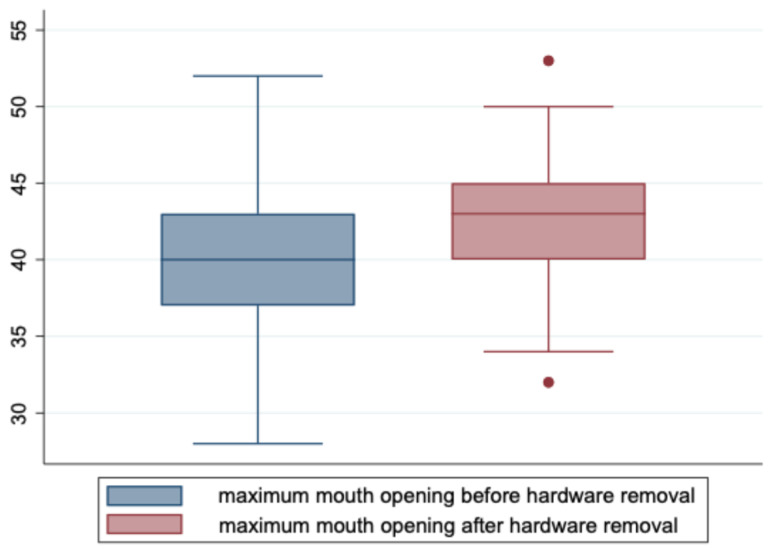
Whisker plot showing function of maximum mouth opening pre- and post removal of osteosynthesis materials (sign test, *p* < 0.001).

The mandibular range of movement before vs. after hardware removal showed clear improvement when applying the classification according to the Helkimo Clinical Dysfunction Index (*p* < 0.001) or the functional classification according to RDC/ TMD (*p* < 0.001) in the context of sign tests (see Table 5). The maximum mouth opening (MMO) also improved significantly (*p* < 0.001) as a result of the second intervention (see Figure 5).

In the extended sample (n sub-sample = 92), compared to Skroch et al.’s study (*n* = 41) (see Table 6), the results for the determination of 3D volumetry according to the methodology of Skroch et al. showed a weak correlation of *p* = 0.047 (regression coefficient B = −0.141; OR = 0.87; 95% CI = [−0.280, −0.002]) between patient age and relative volumetric changes in the period between initial osteosynthetic treatment (T1) and the second intervention for removal of osteosynthesis material (T2) [12]. According to the definitions by Zieliński and Gawda, this describes a very small effect [26].

## 4. Discussion

This study aims to evaluate potential factors affecting intracapsular scarring after ORIF and any possible implications regarding osteosynthesis material removal.

Intracapsular scarring of various degrees was found in three out of four patients of our sample at T2 with RMI. For this reason, our study aimed to examine its causative factors. Skroch et al. assumed that scarry adhesions in the temporomandibular joint may be responsible for osseous resorption within the scope of condylar remodelling after ORIF of CHFs [12]. From orthopaedics, it is well known that fibrous adhesions can also lead to joint stiffness [29]. Although ORIF provides a better functional outcome in comparison to conservative treatment [7,30], some papers already alluded to the risk of functional restrictions after ORIF due to intracapsular scarring [12,15].

To the best of our knowledge however clinical data on the development and effect of intra-articular scarring in the temporomandibular joint after surgical treatment of condylar head fractures are not described in the literature yet. According to our own previous work, functional effects on disc mobility and functional range, as well as on degenerative resorption after ORIF in CHFs, were assumed [12,13]. The necessity to remove metallic implants, which can be derived from previous observations, is currently the subject of controversial scientific debate [31,32,33,34]. To the best of our knowledge, the intra-articular scarring presented in the study at hand, as clinically objectified by our working group, i.e., in the context of a second surgical procedure following initial surgical treatment of a mandibular condylar head fracture, has not yet been reported on by other working groups. Smolka et al., Neuhaus et al., and Park et al., who do not routinely remove metallic implants, argue that any risks associated with osteosynthesis material remaining in place, in the absence of symptoms, do not justify incurring the risk of a second procedure [31,32,34]. Nevertheless, the authors concede exceptions for loosening osteosynthesis material, including the dislocation of the fragments or incompatibility of the osteosynthesis material [31,32]. Recently, there was another report on absent negative effects on MMO in a smaller patient sample of 32 CHF where metallic implants remained in place [34]. However, the assessment of mouth opening alone may not be suited as a single parameter concerning functional limitations of the mandibular range of motion. Since 1995, the senior author has routinely performed metal removal after ORIF in CHFs, and regularly noted some localised or pronounced scarring, even when radiological and functional findings were unremarkable [12]. According to the papers of our working group preceding this study by Skroch et al. and Kolk et al., a possible correlation between the type of osteosynthesis material and intra-articular scarring was assumed, which could not be confirmed in the expanded sample of the present study [12,13]. As already shown by Skroch et al. for resorptions in the run of postoperative condylar remodelling [12], the complexity of the fracture has an effect on intra-articular scarring. The present study shows that major fragmentation incurs an increased probability of localised scarring in the temporomandibular joint (*p* = 0.029; OR = 3.182; medium effect size) [26]. According to the study by Skroch et al., major fragmentation also tends to increase volume resoption [12], and, according to the observations of the present study, scarring often occurs in the area of the earlier fracture gap. It can therefore reasonably be assumed that major fragmentation with multiple fracture gaps and defect zones will induce more extensive scarring, while the phenomenon of scarring starting from the former fracture gap can generally also be observed in fractures with non-minor or major fragmentation. This may result in immobilisation of the disc and frequently also more pronounced resorption in the area of the surface of the condylar head [12,15]. In this respect, the present study was able to statistically confirm previous observations and hypotheses on pathophysiology. The fact that an unstable occlusion will affect the physiological function of the temporomandibular joint was already investigated in detail in several studies, particularly on temporomandibular dysfunctions (TMDs) and CHFs [20,21,35,36]. Kolk et al. showed that CHF patients over 25 years of age or with loss of posterior dental support were prone to TMDs in the case of conservative treatment [13]. In the absence of dental support, the fractured condylar head and TMJ, respectively, are exposed to an increased dorsal load vector, particularly due to the traction of the lateral pterygoid muscle, which cannot be compensated for dentally [37]. This imbalance and the decrease in posterior supporting zones on the fracture side is likely to promote a tendency for localised scar formation in the TMJ capsule (*p* = 0.07; OR = 3.36; medium effect size) [26]. In CHFs that underwent ORIF without sufficient biomechanical stabilisation, this loss of posterior supporting zones could even lead to secondary displacement and/or pseudarthrosis, as CHFs are exposed to high alternating loads in the sense of tensile, rotational, or shear forces, especially in cases with unstable occlusion [38]. Fracture age is identified in the present study as a potential risk factor for pronounced scarring (*p* = 0.018). However, as the effect size was very small (OR = 1.113) [26], fracture age as a risk factor for pronounced scarring needs to be interpreted with caution and requires further investigation in future research. In a recent study by Pienkohs et al., a possible correlation between the fracture age of unilateral and bilateral condylar head fractures and the duration of surgery was reported for the first time [16]. It was shown that each day elapsed after the initial trauma translated into an increase in operating time of approximately five minutes, on average [16]. Pienkohs et al. also found that the subjective muscle resistance of the lateral pterygoid muscle increased with the delay between trauma and ORIF [16]. In addition, Kozakiewicz et al. have shown that the contralateral range of motion worsened significantly with delayed surgical treatment of CHFs (*p* < 0.05) [6]. According to these authors, the earliest possible timing of ORIF, as well as reducing the duration of surgery and a smaller number of drill holes, may improve function more quickly [6,16]. Our findings with regard to fracture age therefore seem to be in line with previous recommendations for the early surgical treatment of condylar fractures [4,39].

To our knowledge however there are no other studies to date specifically investigating the influence of fracture age on intra-articular scarring in fractures of the condylar head. At the same time, another study recommended a kind of “wait-and-watch” policy with subsequent re-osteotomy for condylar head fractures, i.e., a late intervention after trauma [40]. Some argue that surgical treatment of bilateral condylar head fractures, even after unsuccessful conservative therapy, constituted an efficient alternative to re-osteotomy for occlusion disorders [40]. Luo et al. also report of ORIF after more than 6 months in unilateral and bilateral condylar head fractures, in which the functional range, in particular MMO and laterotrusion, improved after the vertical ramus height was restored [41]. However, this is diametrically opposed to the results of the present study with regard to scarring, as well as the observations in our own sample and in other studies, e.g., in orthopaedics, regarding the risk of osseous complications and heterotopic ossifications [42,43,44]. Sweeney et al. already assumed that a previous history of non-surgical treatment as well as delayed surgical treatment might lead to a higher risk of heterotopic ossifications due to prolonged inflammatory processes [43]. Furthermore, according to the present study, there is an increased propensity towards generalised scarring and pronounced scar-related limitations in the traumatised temporomandibular joint in the case of osseous changes within the meaning of heterotopic ossifications and/or osteophytes (*p* = 0.027), substantiated by a medium close to large effect size (OR = 4.171) [26]. According to our study’s data, the probability of subtotal fusion in the dorsal recess or in the lower joint space of the fractured temporomandibular joint increases by around 17% (OR = 1.167) with each day elapsing between initial trauma and surgical treatment. For this reason, an early surgical treatment seems favourable, which is also economically worthwhile due to the shorter duration of surgery [16]. However, due to the very small to small effect size, further investigation is needed before giving clinical advice [26]. Nevertheless, the findings of the present study basically support the recommendations regarding optimal time for surgery previously published by our working group [16]. Relationships between scarring and categorisation of functionality according to the Helkimo Clinical Dysfunction Index and RDC/TMD according to John et al. could not be statistically verified to date, nor regarding localised scarring (Helkimo *p* = 0.422; RDC/TMD *p* = 0.594), or regarding pronounced scarring (Helkimo *p* = 0.410; RDC/TMD *p* = 0.350) [17,18]. However, numerical categorisation of the functional parameters and assessment of MMO, protrusion, and latero- and mediotrusion [in mm] revealed significant results in the present study. For example, pronounced scarring in the surgically treated temporomandibular joint capsule was significantly more likely for laterotrusion ≤ 3 mm (*p* = 0.016). With an OR = 6.086, the effect size was large, highlighting a strong effect warranting potential clinical relevance [26]. Although statistically significant, the effect of localised adhesions on mandibular function was very small, specifically concerning the range of mediotrusive movement (≤5 mm; *p* = 0.013; OR = 0.236) [26]. Therefore, intra-articular scarring may play a decisive role in patients with limited laterotrusion following ORIF of CHFs; however, further investigations regarding mediotrusion in patients presenting with localised scarring are needed. Early secondary surgery for the restoration of functional joint anatomy and simultaneous removal of osteosynthesis material should be considered in cases with more pronounced limitations, particularly for the purpose of scar adhesiolysis, and may have a positive effect on mandibular function, as presented in our sample (*p* < 0.001, D_i_ before and after adhesiolysis; and *p* < 0.001, RDC/TMD before and after adhesiolysis; sign tests, each). Scarring in joints may not only impair function in the long term but may also not at least cause pain [14,45]. In joint surgery in other disciplines, such as in orthopaedics and trauma surgery, secondary procedures to release fibrous adhesions are an established standard, e.g., after surgical treatment of knee trauma, in order to avoid possible postoperative stiffness [14,46,47,48].

The present study presents several limitations. According to the data collected, the function—in particular the ranges of laterotrusion and mediotrusion under objective GETA conditions—improved significantly (*p* < 0.001) after scar adhesiolysis compared to the clinical range of function recorded at the time before removal of the metallic implant (see Table 2). Due to expected drop-outs, the function was assessed in the context of the preoperative patient education for the second procedure or immediately afterwards. Postoperative function, on the other hand, was measured under objective conditions in GETA. Therefore, the long term development or maintenance of functionality was not recorded in this study, which represents a significant limitation of the study. A medium-term follow-up examination of functionality, at least 6 months after surgery in further studies, is therefore to be recommended.

In addition, the functional impairments following surgical treatment of condylar head fractures may be outcomes of multifactorial influences. Apart from intra-articular scarring, the patient’s adherence to a training regime exercising their range of motion also plays an important role [15]. This factor could not be taken into account in the present analysis and requires more detailed investigation in further studies.

With *p* = 0.047 and a very small effect size (OR = 0.87), the present review was only conditionally able to support the causal connection as presented by Skroch et al. (Skroch: *p* = 0.011) between patient age and relative volumetric transformation after ORIF for our sample [12,26]. The negative linear regression coefficient (B = −0.141) for relative volumetric change [%] in relation to patient age indicates that with increasing age, the overall condylar volume changes in the course of postoperative remodelling are less pronounced in older than in younger patients. This is consistent with the observations of Lindahl et al, i.e., that the ability of remodelling is more functionally adaptive by adulthood than a potential restitutio ad integrum in young patients [49]. A causal connection as observed and presented by Skroch et al. between the complexity of the fracture and condylar remodelling (according to Skroch: *p* < 0.001) could no longer be statistically verified in the present study, i.e., in the extended sample [12]. Further studies are therefore necessary to investigate the influence of the degree of fragmentation on osseous volumetric changes in the mandibular condylar head.

A further limitation of our study, in addition to the basically retrospective design of evaluation (although data were prospectively collected here), is the subjective classification of the degree of intra-articular scarring (localised scarring, pronounced scarring with limitations, and fusion of the dorsal recess and lower joint compartment, respectively) by intraoperative visual and haptic assessments by the senior author (A.N., active in the treatment of CHFs for 30 years), documented at the time of the removal of the metallic implant. An advantage of this examination is the likelihood of a high standard of assessment, while subjective, due to the special expertise of the surgeon, the disadvantage being that an objective assessment or differentiated perspectives by different surgeons is not available in this study. In follow-up studies, an objective standard for categorising the degree of scarring should be developed or applied, for example, and supported by additional MRI datasets.

In our opinion, a limitation of the present study, which should not be underestimated, is that potentially relevant factors affecting scar formation could not be objectively recorded, such as possible non-compliance of patients undergoing jaw exercises on their own or the quality of physiotherapy. From a statistical point of view, the relatively small number of cases (*n* = 80 patients or 96 fractures) also limits the significance of the study, particularly with regard to the correlation between osseous changes (*n* = 8, present in 9.6% of the cases) and pronounced scarring (present in 21.7%). It is therefore recommended that the correlation between osseous changes and pronounced intracapsular scarring be examined in further studies with a larger case number.

A retroauricular approach was chosen for all patients, which allows access to the fracture via the dorsal recess without iatrogenically detaching the lateral capsule attachment from the condylar pole, which is important for disc and joint mobility. Direct comparison with functional data from CHFs that may have been treated via a classic preauricular approach is therefore only possible with reservations, as this would require an identical ORIF protocol per our department’s standards. Extra-articular or periarticular scarring as a result of the surgical approach to the joint was not taken into account in this study. Establishing a possible effect of the type of approach taken on intra-articular scarring in the temporomandibular joint itself would require a randomised study with regard to the approach. Thus, a further limitation of this study lies in the fact that this is a single-centre study; surgical procedures may differ from other treatment centres.

Our department routinely performs standardised ORIFs via a retroauricular approach (constant surgeon A.N.) and surgically treats complex CHFs (major and minor fragmentations, i.e., including comminuted fractures). This study contains the largest published sample of CHFs treated via ORIF to date, including subsequent follow-up and 3D analysis of the volumetric changes in the condyle between T1 and T2 based on CBCT and CT data. As the removal of osteosynthesis material is not routinely performed at other centres, the present study also investigates intracapsular scarring after ORIF clinically, as no previous study has done so to our knowledge and illustrates the functional benefit of a second procedure for adhesiolysis and RMI, even in asymptomatic patients.

Ultimately, multicentre studies with larger patient populations, standardised surgical protocols with regard to the osteosynthesis materials used (e.g., titanium vs. resorbable), with longer observation periods, and with a control cohort without a second procedure for removal of the metallic implant and scar adhesiolysis are necessary in order to make specific recommendations for treatment of intracapsular scarring after ORIF of condylar head fractures. However, the available results suggest that patients after ORIF of joint fractures can benefit from targeted scar removal after ORIF.

## 5. Conclusions

This study demonstrates that patients with pronounced scarring in the lower joint space or dorsal recess post ORIF of CHFs are significantly more likely to exhibit severe functional limitations (*p* = 0.016; OR = 6.806; large effect size) and osseous degenerative changes (*p* = 0.041; OR = 4.171; medium effect size) [26]. The complexity of CHFs (major fragmentation (*p* = 0.029; OR =3.182) vs. minor (*p* = 0.658; OR = 0.805), respectively) and missing dental support (*p* = 0.022; OR = 3.36) can be considered as relevant factors for intra-articular scarring after ORIF (both significant findings present medium effect sizes [26]).

An early surgical treatment of CHFs may be associated with a reduced risk of heterotopic ossification and/or formation of osteophytes (*p* = 0.041; OR = 4.171; medium to large effect size), and lower risk of limitations associated with postoperative scarring (*p* = 0.018; OR = 1.13) [26]. However, due to the very small effect size, this finding needs to be interpreted with caution, warranting further investigation.

Our findings highlight the potential adverse effects of intra-articular scarring after ORIF in CHFs such as functional limitations and osseous degenerative changes and speak in favour of targeted scar adhesiolysis during hardware removal (in our sample, approximately 3–4 months) post ORIF, especially for patients presenting with functional limitations. Further studies with longer follow-ups are recommended to record the function of surgically treated condylar head fractures in the medium-to-long term, based on a larger patient cohort to identify other potential factors promoting scar formation. In particular, patient-dependent risk factors such as adherence to consistent jaw mobility training should be evaluated in future studies.

## Figures and Tables

**Table 1:** Characterisation of sample of condylar head fractures with eligible datasets.

	Fractures *n* = 96	Patients *n* = 80	Mean Age in Years (SD)	Median	Gender (% of Fractures)
Unilateral Fractures	64	64	44.9	48.5	Male 45.3%
			(19)		Female 54.7%
Bilateral Fractures	32	16	48.8	48	Male 43.75%
			(19)		Female 56.25%
Major Fragmentation	28	26	51.6	51	Male 32.1%
			(20)		Female 67.9%
Minor Fragmentation	23	23	47.1	49	Male 39.1%
			(19)		Female 60.9%
Non-fragmented	45	39	42.1	48	Male 55.6%
			(19)		Female 44.4%

**Table 2:** Logistic associations (regression), dependent variable: localised scarring (* *p* < 0.05). OR ≥ 1.44 indicates small effect, OR ≥ 2.48 indicates medium effect, and OR ≥ 4.27 indicates large effect [26].

Predictor	*p*	OR with 95% CI
Patient age	0.869	0.998 [0.977, 1.020]
Gender (female)	0.474	0.73 [0.312, 1.717]
Concomitant fractures	0.073	1.8 [0.192, 1.075]
Dental support zone missing	0.022 *	3.36 [1.191, 9.459]
Screw protrusion (T2)	0.536	1.31 [0.554, 3.108]
Osteosynthesis loosening (T2)	0.079	2.24 [0.910, 5.506]
Number of microplates (T2)	0.572	1.108 [0.777, 1.581]
Major fragmentation	0.029 *	3.182 [1.123, 9.013]
Minor fragmentation	0.658	0.805 [0.308, 2.103]
Fracture age	0.390	0.965 [0.891, 1.046]
Osseous alteration (T2)	0.458	0.592 [0.148, 2.361]
Months between initial surgery and RMI	0.374	0.90 [0.717, 1.133]

**Table 3:** Logistic associations (regression), dependent variable: pronounced scarring (* *p* < 0.05). OR ≥ 1.44 indicates small effect, OR ≥ 2.48 indicates medium effect, and OR ≥ 4.27 indicates large effect [26].

Predictor	*p*	OR with 95% CI
Patient age	0.419	1.01 [0.984, 1.039]
Gender (female)	0.603	1.347 [0.438, 4.138]
Concomitant fractures	0.201	2 [0.691, 5.787]
Dental support zone missing	0.071	0.344 [0.108, 1.097]
Screw protrusion (T2)	0.387	0.602 [0.191, 1.897]
Osteosynthesis loosening (T2)	-	1 [n.a.]
Number of microplates	0.103	1.460 [0.927, 2.302]
Major fragmentation	0.472	0.643 [0.193, 2.144]
Minor fragmentation	0.435	0.58 [0.148, 2.275]
Fracture age	0.018 *	1.113 [1.018, 1.216]
Osseous alteration (T2)	0.041 *	4.171 [1.058, 16.452]
Months between initial surgery and RMI	0.254	1.128 [0.917, 1.389]

**Table 4:** Logistic associations between postoperative function (D_i_ and RDC/TMD) and dependent variables: localised and pronounced scarring. OR ≥ 1.44 indicates small effect, OR ≥ 2.48 indicates medium effect, and OR ≥ 4.27 indicates large effect [26].

Dependent Variable	Predictor	*p*	OR with 95% CI
	D_i_	0.422	0.778 [0.421, 1.436]
Localised scarring	RDC/TMD	0.594	0.847 [0.460, 1.559]
	D_i_	0.410	1.445 [0.603, 3.464]
Pronounced scarring	RDC/TMD	0.350	1.488 [0.647, 3.424]

**Table 5:** Crosstab between pre- and postoperative function according to Helkimo (sign test, *p* < 0.001) and RDC/TMD (sign test, *p* < 0.001).

Preoperative D_i_			Postoperative D_i_		Total
	**0**	**1**	**2**	**3**	
0	21	2	0	0	23
1	11	5	0	0	16
2	10	2	0	0	12
3	2	1	0	0	3
Total	44	10	0	0	54
**Preoperative RDC/TMD**			**Postoperative RDC/TMD**		**Total**
	**0**	**1**	**2**	**3**	
0	3	2	21	0	27
1	2	3	19	0	23
2	1	2	11	0	14
3	0	0	2	0	2
Total	6	7	53	0	66

**Table 6:** Linear associations (regression), *n* = 92, dependent variable: relative volumetric change (* *p* < 0.05).

Predictor	Regression Coefficient B with 95% CI	*p*
Patient age	−0.141 [−0.280, −0.002]	0.047 *
Major fragmentation	n.a. [−9411, 1757]	0.176
Female patient	−3.576 [−8.483, 1.331]	0.151
Osseous alteration	2.338 [−18.921, −13.665]	0.570

## Data Availability

The data presented in this study are available on request from the corresponding author.
